# Bronchoalveolar Tregs are associated with duration of mechanical ventilation in acute respiratory distress syndrome

**DOI:** 10.1186/s12967-020-02595-3

**Published:** 2020-11-11

**Authors:** Dustin L. Norton, Agathe Ceppe, Miriya K. Tune, Matthew McCravy, Thomas Devlin, M. Bradley Drummond, Shannon S. Carson, Benjamin G. Vincent, Robert S. Hagan, Hong Dang, Claire M. Doerschuk, Jason R. Mock

**Affiliations:** 1 Division of Pulmonary Diseases and Critical Care Medicine, University of North Carolina, Chapel Hill, NC USA; 2 Department of Medicine, University of North Carolina, Chapel Hill, NC USA; 3 Marsico Lung Institute, University of North Carolina, Chapel Hill, NC USA; 4 Department of Respiratory Care, University of North Carolina, Chapel Hill, NC USA; 5 Department of Microbiology and Immunology, University of North Carolina, Chapel Hill, NC USA; 6 Division of Hematology/Oncology, University of North Carolina, Chapel Hill, NC USA; 7 Present Address: Section of Pulmonary, Critical Care, Allergy and Immunologic Diseases, Wake Forest School of Medicine, Winston-Salem, NC USA; 8 Present Address: Division of Pulmonary, Allergy, and Critical Care Medicine, Duke University School of Medicine, Durham, NC USA; 9 Division of Pulmonary Diseases and Critical Care Medicine, Department of Medicine, University of North Carolina School of Medicine, Marsico Hall 7203, 125 Mason Farm Road, Chapel Hill, NC 27599 USA

**Keywords:** Acute respiratory distress syndrome, Regulatory T cells, Resolution

## Abstract

**Background:**

Foxp3^+^ regulatory T cells (Tregs) play essential roles in immune homeostasis and repair of damaged lung tissue. We hypothesized that patients whose lung injury resolves quickly, as measured by time to liberation from mechanical ventilation, have a higher percentage of Tregs amongst CD4^+^ T cells in either airway, bronchoalveolar lavage (BAL) or peripheral blood samples.

**Methods:**

We prospectively enrolled patients with ARDS requiring mechanical ventilation and collected serial samples, the first within 72 h of ARDS diagnosis (day 0) and the second 48–96 h later (day 3). We analyzed immune cell populations and cytokines in BAL, tracheal aspirates and peripheral blood, as well as cytokines in plasma, obtained at the time of bronchoscopy. The study cohort was divided into fast resolvers (FR; n = 8) and slow resolvers (SR; n = 5), based on the median number of days until first extubation for all participants (n = 13). The primary measure was the percentage of CD4^+^ T cells that were Tregs.

**Results:**

The BAL of FR contained more Tregs than SR. This finding did not extend to Tregs in tracheal aspirates or blood. BAL Tregs expressed more of the full-length FOXP3 than a splice variant missing exon 2 compared to Tregs in simultaneously obtained peripheral blood.

**Conclusion:**

Tregs are present in the bronchoalveolar space during ARDS. A greater percentage of CD4^+^ cells were Tregs in the BAL of FR than SR. Tregs may play a role in the resolution of ARDS, and enhancing their numbers or functions may be a therapeutic target.

## Background

Acute respiratory distress syndrome (ARDS) is a clinical syndrome characterized by a marked inflammatory response within the alveolar space, resulting in rapid-onset of bilateral pulmonary infiltrates and acute respiratory failure [1]. ARDS continues to account for 10% of intensive care unit (ICU) admissions, and in-hospital mortality can be as high as 46% in its most severe form [2]. Understanding mechanisms of resolution of acute lung injury (ALI) is necessary to inform interventions and improve outcomes in ARDS.

Foxp3^+^ regulatory T-cells (Tregs) are a population of CD4^+^ lymphocytes shown to suppress and down-regulate immune responses [3, 4]. Tregs have essential roles in both health and disease, having both protective effects in multiple disease states (autoimmune disease, inflammatory bowel disease, and ANCA vasculitis) and deleterious effects on immune regulation in cancer [5–7]. In experimental models of ALI, Tregs increase following lung injury and play an important role in resolution by suppressing inflammation, promoting epithelial and endothelial cell repair, and reducing fibrosis [8–12]. Tregs are present in the bronchoalveolar compartment in humans with ARDS [9, 13–15], though their kinetics and role during recovery following ARDS in humans have yet to be defined.

ARDS is characterized by rapidly changing kinetics; a single snapshot in time is unlikely to be sufficiently informative. Steinberg et al. first reported the kinetics of alveolar immune cells over 25 years ago, and their work highlighted the neutrophilic predominance in early ARDS [16]. Their work showed that sustained neutrophilia in the bronchoalveolar lavage (BAL) portended a worse prognosis, while alveolar macrophages increased over time in survivors [16]. They also demonstrated that specific inflammatory cytokines correlated with outcomes, but this correlation was dependent on the timing of the bronchoscopy and was not consistent across time points [17].

Few studies have since utilized serial bronchoscopy to evaluate the alveolar space in ARDS. Serial assessments of Tregs in experimental lung injury has provided some indications that these cells may play a role in the resolution of ALI [9, 14]; however, single time point sampling in humans suggests a correlation between Tregs and increased mortality [13]. Serial bronchoscopy will allow better understanding of the heterogeneity of ARDS, of immune cell and mediator kinetics over time, and of determinants of resolution—each of which may be fruitful to discover new therapeutic targets for the treatment of ARDS.

The purpose of this study was to determine if the percentage of Tregs in humans changes during recovery from ARDS and to evaluate associations between the percentage of Tregs in the BAL and the rate of recovery from ARDS. We hypothesized that ARDS patients who resolve quickly (fast resolvers (FR)), will have a higher percentage of CD4^+^ T cells that are Tregs compared to slower resolvers (SR) in BAL, tracheal aspirate and/or peripheral blood. We prospectively enrolled patients with ARDS requiring mechanical ventilation and collected BAL samples soon after admission (day 0) and at day 3. We analyzed immune cell populations and cytokine concentrations in BAL cells and lavage fluid, along with cells and fluid obtained from tracheal aspirates and peripheral blood at the time of bronchoscopy. The study cohort was divided into FR (n = 8) and SR (n = 5) based on the median number of days until extubation for all participants (median = 6 days). The primary measure was the peak percentage of CD4^+^ cells that are FOXP3^+^ (Tregs) in the BAL immune cell population during ARDS. We also measured the changes of other immune cell populations.

## Methods

### Study population

Adult patients ages 18 to 80 admitted to the intensive care unit who meet Berlin criteria for ARDS and required mechanical ventilation were screened for enrollment December 1, 2017 through November 1, 2019 [1]. The University of North Carolina School of Medicine Institutional Review Board approved this study. Authorized representatives provided informed consent if participants were unable to consent. Exclusion criteria included hematologic malignancy, recent chemotherapy, HIV infection, pregnancy, incarceration, an endotracheal tube with an internal diameter of less than 7 mm, and inability to undergo bronchoscopy within 72 h of diagnosis. Exclusion criteria for bronchoscopy included an INR > 3, therapeutic oral anticoagulation, platelets < 50,000 per microliter blood, acute ischemic heart disease or critical cardiac dysrhythmias, refractory hypotension, known or suspected elevation of intracranial pressure, or requirement of a fraction of inspired oxygen (FiO_2_) greater than 90%. Enrolled patients underwent bronchoscopy and BAL with instillation of sterile saline on the day of enrollment (day 0, within 72 h of diagnosis) and 2–4 days later (day 3) if they remained on mechanical ventilation. BAL fluid from two lung segments, peripheral blood, and a tracheal aspirate were collected at the same time.

Baseline clinical data (age, race, gender, comorbidities, etiology of ARDS), and clinical data (illness severity scores, labs, vital signs, ventilator parameters, etc.) were collected. Clinical outcomes were recorded, and groups of fast resolvers (FR) and slow resolvers (SR) were defined based on the median number of days to extubation from mechanical ventilation in the cohort.

### Bronchoscopy protocol

Patients meeting enrollment criteria for the study were assessed at each time point for the appropriateness of bronchoscopy. The procedure was deferred at that time point if the endotracheal or tracheostomy tube had an internal diameter of fewer than 7 mm or if the patient had any of the exclusion criteria described above. Deferment of the procedure also occurred if the clinical team planned for extubation within 12 h.

Patients were pre-oxygenated with 100% oxygen for at least 5 min before and during the procedure. Ventilation was performed in a volume mode with pressure limits of at least 80 cm H_2_O to allow adequate ventilation around the bronchoscope, while positive end-expiratory pressure (PEEP) and tidal volume (V_T_) were left at pre-procedure levels. BAL was performed by passing an Olympus BF-P160 or P180 flexible bronchoscope through the endotracheal or tracheostomy tube and advancing to a wedged position in a segmental or subsegmental bronchus. Segment selection was at the discretion of the research team; however, preference was given to the anterior upper lobe segments, right middle lobe, and lingual, given their preferred location in the supine patient. After the bronchoscope was wedged in the desired segment, aliquots of 50–60 mL of sterile 0.9% sodium chloride (Hospira, Inc., Lake Forest, IL) were instilled in each segment. Retrieval was performed via gentle manual suction until a total return of at least 30 mL was achieved from each segment with a maximum instillation of 180 mL per segment. After the lavage, the appropriate positioning of the endotracheal tube was confirmed, and the bronchoscope was removed. Patients were returned to their prior mode of ventilation, and the FiO_2_ was weaned as tolerated.

### Sample processing

BAL was collected in 50 mL conical tubes, and samples were centrifuged at 400×*g* for 10 min at 4 °C. The supernatant was removed and stored in 1 mL aliquots at − 80 °C. The remaining cellular pellet underwent lysis of red blood cells by adding 20 mL of distilled water with gentle vortexing and then immediately adding 25 mL of phosphate-buffered saline (ThermoFisher Scientific). Then, BAL samples were centrifuged again at 400×*g* for 10 min at 4 °C. The supernatant was aspirated and discarded. The cell pellet was resuspended in 1 mL Media A (480 mL RPMI, 5 mL Pen/Strep, 5 mL 200 mM l-Glutamine, 5 mL 100 mM Sodium pyruvate, and 5 mL 1 M HEPES buffer, Life Technologies). The BAL processing was performed as previously described [18].

Tracheal aspirates were collected in sterile sputum containers and weighed. Media A was added at a rate of 8 mL per gram of tracheal aspirate weight and vortexed at low to medium speed for 30 s. The sample was then filtered through 100 µm nylon mesh filters (BD Bioscience) into a new 50 mL conical tube. Filtered samples were centrifuged at 400×*g* for 5 min at 4 °C. Supernatant was removed and stored in 1 mL aliquots at − 80 ºC, and the cell pellet was resuspended in 1 mL of Media A.

Peripheral blood was collected in two 6 mL EDTA tubes (BD Bioscience), and tubes were inverted 6–12 times after collection to ensure appropriate mixing. Samples were centrifuged at 4 °C at 1000×*g* for 10 min with the acceleration and brake off. The upper plasma layer was gently removed and transferred to a fresh 15 mL polypropylene tube. The buffy coat was then removed with a P1000 pipet and decanted into a 50 mL conical. The plasma was recentrifuged to eliminate residual white blood cells at 1500×*g* at 4 °C for 10 min. The plasma layer was removed and stored in 1 mL aliquots at − 80 °C. The cell pellet from the recentrifuged plasma was resuspended and added to the buffy coat. Next, the cell pellet underwent lysis of red blood cells by adding 20 mL of distilled water with gentle vortexing and then immediately adding 25 mL of phosphate-buffered saline (ThermoFisher Scientific). Then, buffy coat samples were centrifuged again at 400×*g* for 10 min at 4 °C. The supernatant was aspirated and discarded, and the cell pellet was resuspended in 1 mL Media A.

The total cell number for each sample was enumerated with a hemocytometer after staining with trypan blue. The cells were then subjected to multicolor flow cytometry, and any excess cells remaining after flow cytometry were cryopreserved using Recovery™ cell culture freezing medium per manufacture protocol (ThermoFisher).

### Flow cytometry

**Fig. 1:**
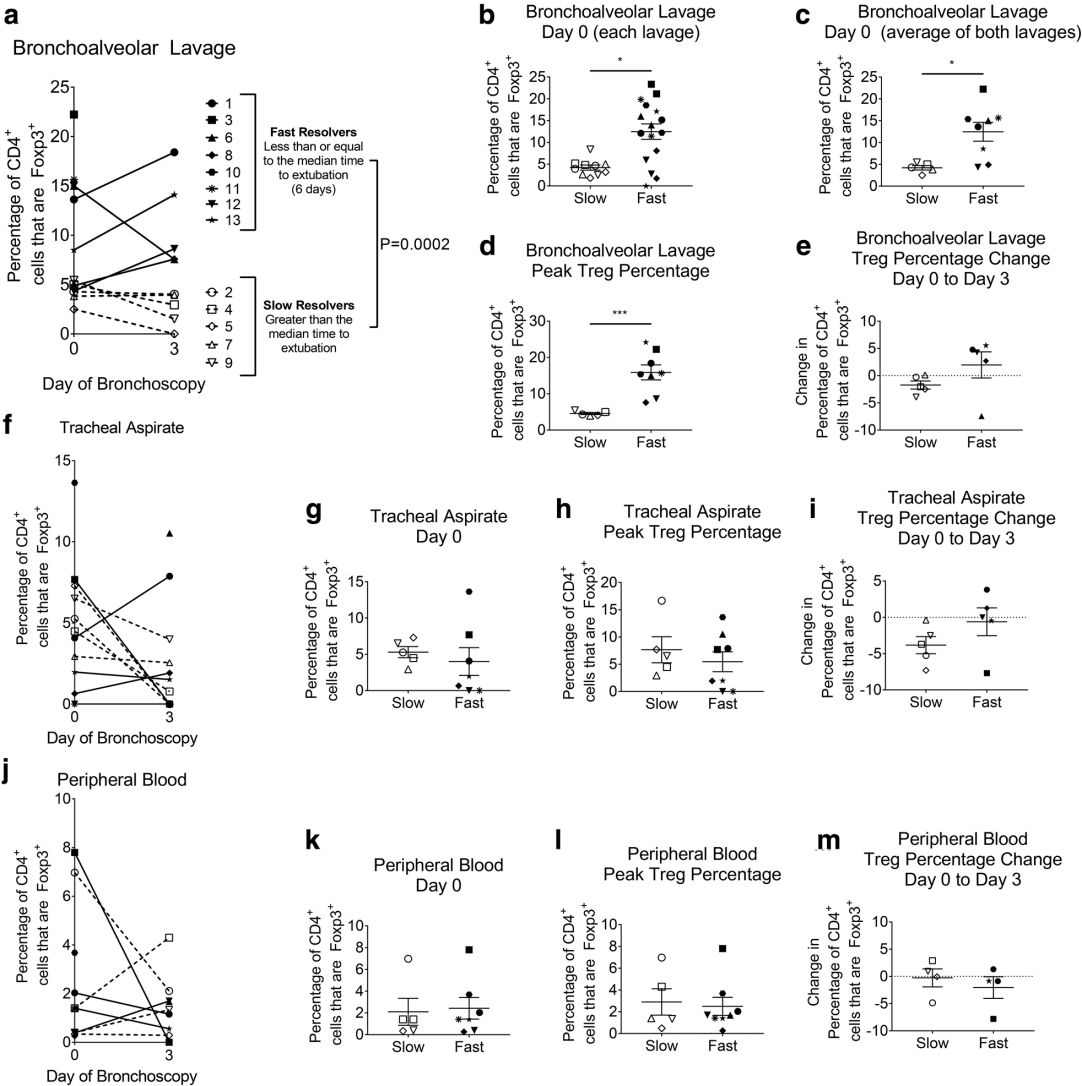
Treg percentage during ARDS in the bronchoalveolar lavage (BAL), tracheal aspirate, and peripheral blood. Percentages of CD4^+^ cells that are FOXP3^+^ were quantified in cells obtained from BAL, tracheal aspirate, or peripheral blood. **a** The mean percentages of CD4^+^ cells that are FOXP3^+^ in two BALs performed during bronchoscopy at two time points (n = 13). Each participant is identified by a unique symbol. Each is categorized as a slow or fast resolver, based on the median number of days to extubation. Slow resolvers are open symbols with a dashed line connecting points, while fast resolvers are filled in solid black with a solid line connecting points. **b** The percentage of CD4^+^ cells that are FOXP3^+^ for each lavage (two for each participant) at Day 0. Participants are categorized as slow or fast resolvers. **c** The average percentage of FOXP3^+^ CD4^+^ cells of the two BALs during bronchoscopy performed on Day 0. Participants are categorized as slow or fast resolvers (SR, n = 5; FR, n = 8). **d** The highest percentage of FOXP3^+^ CD4^+^ cells obtained at either time point is shown for slow and fast resolvers (SR, n = 5; FR, n = 8). **e** The change in average percentages of FOXP3^+^ CD4^+^ cells between Day 0 and Day 3; participants are categorized as slow or fast resolvers (SR n = 5; FR n = 5). Of note, the lower FR number is because 3 FR were extubated before the second bronchoscopy time point. **f** The percentages of CD4^+^ cells that are FOXP3^+^ in tracheal aspirates obtained just prior to bronchoscopy at Day 0 and 3 (Day 0, n = 12; Day 3, n = 11). **g** The percentage of FOXP3^+^ CD4^+^ cells in tracheal aspirates at Day 0. Participants are categorized as slow or fast resolvers (SR, n = 5; FR, n = 7). **h** The highest percentage of FOXP3^+^ CD4^+^ cells in tracheal aspirates obtained at either Day 0 or Day 3; participants categorized as slow or fast resolvers (SR, n = 5; FR, n = 8). **i** The change in average percentages of FOXP3^+^ CD4^+^ cells in tracheal aspirates between Day 0 and Day 3; participants grouped as slow or fast resolvers (SR, n = 5; FR, n = 5). **j** The percentages of CD4^+^ cells that are FOXP3^+^ in peripheral blood obtained at the time of bronchoscopy at two time points (Day 0, n = 12; Day 3, n = 9). **k** The percentage of FOXP3^+^ CD4^+^ cells in peripheral blood at Day 0. Participants are categorized as slow or fast resolvers (SR, n = 5; FR, n = 7). **l** The highest percentage of FOXP3^+^ CD4^+^ cells in peripheral blood at either time point, participants categorized as slow or fast resolvers (SR, n = 5; FR, n = 8). **m** The change in average percentages of FOXP3^+^ CD4^+^ cells in peripheral blood between Day 0 to Day 3, participants categorized as slow or fast resolvers (SR, n = 4; FR, n = 4). Data are expressed as the mean ± standard error of the mean. *P < 0.05, ***P < 0.001

**Fig. 2:**
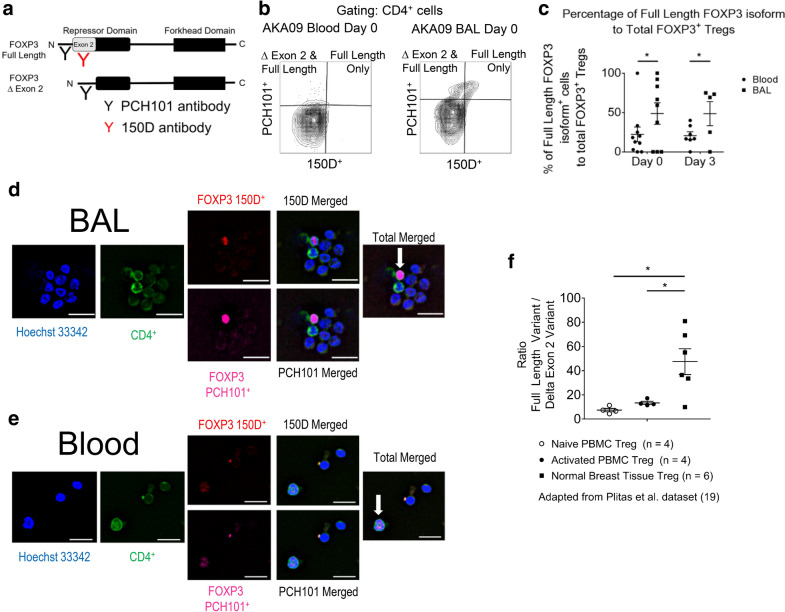
*FOXP3* mRNA splicing differs in Tregs from the BAL compared to the peripheral blood. **a** Schematic of two antibody clones that distinguish two of the most common FOXP3 isoforms. Both antibodies (clones PCH101 and 150D) bind to the full-length protein, whereas clone 150D does not bind to the splice variant missing region of FOXP3 coded by exon 2, part of the repressor domain of FOXP3. The regions where two FOXP3 monoclonal antibodies, 150D and PCH101, bind to the FOXP3 protein are illustrated. **b** Flow cytometric plots and gating schemes used to identify the full length FOXP3 and the splice variant of FOXP3 are illustrated for Tregs in the peripheral blood or BAL using a set of stored blood and BAL cell samples. Gating and dot plot results are representative of at least three independent experiments. **c** Percentage of full length FOXP3 isoform^+^ cells to total FOXP3^+^ Tregs isolated from either the peripheral blood or BAL samples (Day 0: n = 9 lavages, blood n = 10 samples; Day 3: n = 5 lavages, blood n = 7 samples). Data were analyzed using a two way repeated measure ANOVA, with both days and type as repeated factors. The blood results are significantly different from the BAL results on both day 0 and day 3 (*P* = 0.0245). The data are not different between days. **d**, **e** Immunofluorescence of BAL (**d**) or peripheral blood (**e**) cytospins prepared from a participant and stained for DNA, CD4^+^, FOXP3^+^ clone 150D, and FOXP3^+^ clone PCH101. Arrows indicate Tregs. White bar: 20 µM. **f** RNA-Seq data examining two *FOXP3* splice variants in a previously reported data set of Tregs isolated from human breast tissue or peripheral blood mononuclear cells (PBMC) [19]. Data expressed as the mean ± SEM.* P* values for the RNA-Seq data determined by paired and independent t-tests. *P < 0.05

**Fig. 3:**
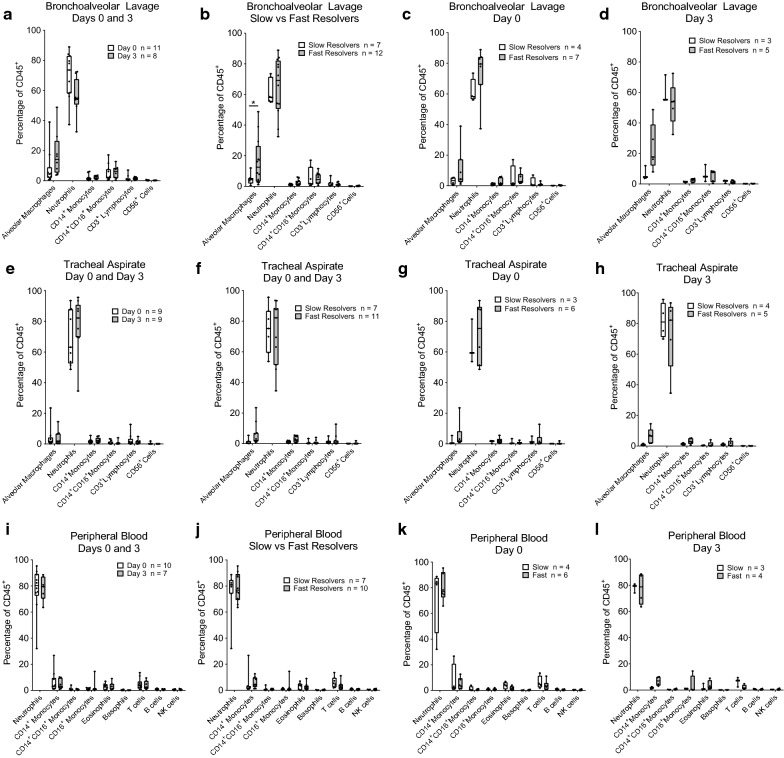
Immune cell subsets identified in the bronchoalveolar lavage (BAL), tracheal aspirate, or peripheral blood by flow cytometry. Box-and-Whisker plots showing median, minimum, and maximum with overlay of individual data points (dots) of immune cells, expressed as a percentage of CD45^+^ cells at day 0 and/or day 3 after enrollment. **a**–**d** BAL immune cells expressed as a percentage of CD45^+^ cells at day 0 or day 3 after enrollment. **a** Percentages are shown for all participants at both time points. **b** Fast and slow responders are compared combining data from both days. **c** Fast and slow responders are compared at Day 0. **d** Fast and slow responders are compared at Day 3. **e**–**h** The tracheal aspirate immune cells expressed as a percentage of CD45^+^ cells at day 0 or day 3 after enrollment. Percentages are shown for participants at both time points (**e**), grouped as fast or slow resolvers (**f**), and categorized as slow or fast resolvers at Day 0 (**g**) or Day 3 (**h**). **i**–**l** The peripheral blood immune cells expressed as a percentage of CD45^+^ cells at day 0 or day 3 after enrollment. Percentages are shown for participants at both time points (**i**), grouped as fast or slow resolvers (**j**) and categorized as slow or fast resolvers at Day 0 (**k**) or Day 3 (**l**). The flow cytometric plots and gating scheme used for the identification of immune cells with gating were adapted from [23]. Data were analyzed per type of cell, without correction of multiple comparisons. For each cell type, repeated measure ANOVA was used to compare day 0 and 3, fast and slow resolvers, and interaction

**Fig. 4:**
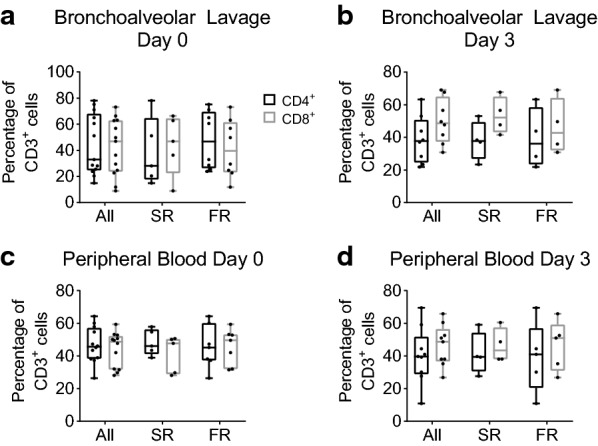
Percentage of CD3^+^ cells that are either CD4^+^ or CD8^+^ in the bronchoalveolar lavage (BAL) or peripheral blood from participants at either Day 0 or Day 3. Box-and-Whisker plots showing median, minimum, and maximum with overlay of individual data points (dots) of the percentage of CD3^+^ cells that are either CD4^+^ or CD8^+^ on day 0 or day 3 after enrollment. **a**, **b** Percentage of CD3^+^ lymphocytes that are either CD4^+^ or CD8^+^ in BAL, shown as the average of two lavages performed during bronchoscopy at Day 0 (**a**) and Day 3 (**b**). The participants are categorized as slow (SR) or fast resolvers (FR) at either Day 0 (SR n = 5; FR n = 8) or Day 3 (SR n = 4; FR n = 4). **c**, **d** Percentage of CD3^+^ lymphocytes that are either CD4^+^ or CD8^+^ in the peripheral blood, shown for all samples at Day 0 (**c**) and Day 3 (**d**). The participants are categorized as slow or fast resolvers at either Day 0 (SR n = 5; FR n = 7) or Day 3 (SR n = 4; FR n = 5). No significant differences were identified. Data were analyzed per cell type, without correction for multiple comparisons. For each cell type, repeated measure ANOVA was used to compare day 0 and 3, fast and slow resolvers, and interaction

1.5 × 10^6^ cells per sample were added fresh to a 96-well plate. 1 µL of Fc receptor block (Biolegend) was added to each sample and allowed to incubate for 10 min at 4 °C. Extracellular antibodies (Additional file 1: Table S1) in cytometry buffer (PBS with 1.5% BSA and 2 mM EDTA) were then added and allowed to incubate on ice for 30 min before washing with 100 µL of buffer. Samples were then centrifuged at 400×*g* for 5 min at 4 °C, and the supernatant was removed. For intracellular staining, the cells underwent fixation and permeabilization with the Foxp3/Transcription Factor Staining Buffer Set (eBioscience, San Diego, CA). Next, fixed and permeabilized single-cell suspensions were incubated on ice for 30 min. Samples were washed with 100 µL of Perm buffer (eBioscience) and centrifuged at 500×*g* for 5 min at 4 °C. The cell pellet was resuspended in 50 µL of perm buffer, followed by the addition of intracellular antibodies (Additional file 1: Table S1), and the sample incubated on ice for 30 min. The sample again was washed with 100 µL of Perm buffer followed by centrifugation at 500×*g* for 5 min at 4 °C. The final cell pellet was resuspended in 100 µL of cytometry buffer and run on a Cytoflex Cytometer (Beckman Coulter Life Sciences) with laser parameters listed in Additional file 1: Table S2. Of note, two participant’s samples were not subjected to flow cytometric analysis before freezing. These two samples (participant 2 and 3) were analyzed several days after freezing, and data from these two participant samples used for the Treg and lymphocyte analysis (Figs. 1, 2, and 4). Data from these two participants were not used in the immune cell population analysis in Fig. 3, given the decrease in neutrophil percentage after freezing.

### Immunofluorescence

Immunofluorescence staining of BAL and peripheral blood cells was performed on cytocentrifuge preparations (StatSpin Cytofuge; Beckman Coulter). Cells were fixed with 10% neutral buffered formalin for 10 min at room temperature and washed 3 times with phosphate-buffered saline. Cells were then permeabilized with PBS + 0.1% Triton X-100 (ThermoFisher Scientific) for 10 min at room temperature. Next, Odyssey blocking buffer (Li-Cor, Lincoln, NE) was added for 30 min by applying the buffer to the slides for 30 min at room temperature. Evaporation was minimized by using a slide moisture chamber (Scientific Device Laboratory, 197-BL). Next, all primary antibodies (Additional file 1: Table S1) were diluted in Odyssey blocking buffer and incubated at 4 °C overnight in a slide moisture chamber. After overnight incubation, the slides were washed three times with PBS and then incubated with Hoechst 33342 (2 µg/mL) in 250 µL of Odyssey blocking buffer for 30 min in a slide moisture chamber. The slides were then washed three times with PBS. Before imaging, the slides were mounted with PBS supplemented with 10% glycerol and then covered with a coverslip. Slides were visualized by immunofluorescence with an Olympus VS120 Virtual Slide Microscope (Olympus Corporation, PA), and OlyVIA software (Olympus) was used for image analysis.

### Immune mediator analysis

The Bio-Plex MAGPIX platform (Bio-Rad, Hercules, CA) was used to measure the levels of 37 cytokines/chemokines in the BAL fluid supernatants using the Bio-Plex Pro Human Inflammation Chemokine Panel 37-Plex kit (Bio-Rad; Catalog #171AL001M) per the manufacturer's protocol.

### Transcriptomic analysis of *FOXP3* splice variants

Previously reported RNA-seq data were examined for the expression of *FOXP3* splice variants in Tregs from different sites [19]. The accession number for the published sequencing data is BioProject: PRJNA350777, and GEO: GSE89225. To obtain transcript isoform data, the selected RNA sequence reads from associated Sequence Read Archive (SRA) were downloaded and converted to FASTQ files using the SRA toolkit (http://ncbi.github.io/sra-tools/ and the SRA Toolkit Development Team). Then the sequence reads were mapped to the reference genome GRCh38 with Gencode v32 gene and transcript annotation using HISAT2 [20], and gene and transcript expression were estimated from the alignment BAM files using StringTie [21]. Next, the fragment per kilobase (fpkm) values for the full-length FOXP3 and the transcript lacking exon 2 were obtained. A ratio of the full-length variant to the transcript lacking exon 2 variant (delta exon 2) was determined. In instances where the fpkm value was 0, then a low value of 0.5 was substituted for evaluation in the ratio calculations.

### Statistics

The primary outcome of interest was the peak Tregs as a percentage of CD4^+^ cells in the BAL during ARDS in slow resolvers (SR) compared to fast resolvers (FR). The classification in SR and FR was defined based on the median of the number of days until the first extubation. The distribution of the number of ventilator days was calculated, and the median number of ventilation days reported. Subjects were then classified into FR (subjects with ventilation days lower and equal to the median) and SR (subjects with ventilation days higher than the median). We also evaluated days to “extubatable ventilator settings” (to assess improvement in lung function and reduce confounding by other variables that affect time to extubation). The participants distributed the same by either method. A comparison of peak Tregs between fast and slow resolvers was made using 2-sided independent t-tests, with the Satterthwaite method. Inclusion of days (day 0 and 3) were analyzed with two-way repeated measure ANOVA, including the interaction term. Comparison of naïve and activated Tregs (Fig. 2f) was made with paired t-tests, while the comparison of normal breast tissue Tregs with either naïve or activated Tregs was made with independent t-tests. Statistical analyses were performed using GraphPad Prism 7 software (La Jolla, CA) and SAS 9.4 (Cary, NC). Other statistical methods are provided in the figure legends. Statistical difference was accepted at P < 0.05.

## Results

### Participant enrollment, characteristics, and sample collections

Mechanically ventilated adults admitted to a medical intensive care unit at a single academic center from December 1, 2017 through November 1, 2019, and who met the Berlin definition of ARDS [1] were considered for an IRB-approved observational research study. Table 1 lists each participant’s course and the number of bronchoscopies performed.

**Table 1:**
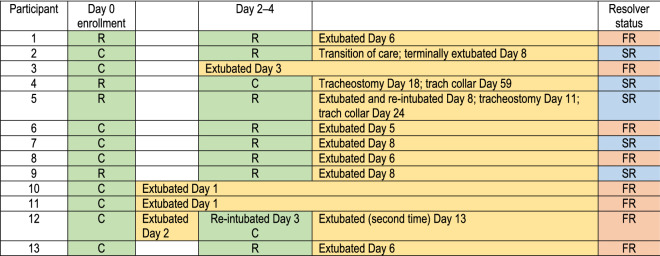
Time to first extubation for each participant and the serial bronchoscopies performed in this study

One participant (participant 12) was extubated after 2 days and subsequently suffered an aspiration event, which required re-intubation. Using the definitions of FR and SR in this study, this participant was classifid as a fast resolver. C = Bronchoscopy was clinically indicated. Excess samples were used for study purposes. R = Bronchoscopy was bronchoscopy performed solely for study. SR = Slow Resolvers; FR = Fast Resolvers

Table 2 displays the 13 participants’ baseline clinical characteristics. The first bronchoscopy was performed within 72 h of diagnosis (Day 0). Ten of these remained intubated 48–96 h later and underwent a second bronchoscopy (Day 3). Nine of the initial 13 bronchoscopies were clinically indicated, and all included two lavages. Two of the repeated bronchoscopies were clinically indicated by the treating ICU team, and all but one of the ten participants underwent two lavages. The mean time to extubation was 10.2 days, and the median was six days. The study cohort was divided into fast resolvers (FR; n = 8) and slow resolvers (SR; n = 5) based on the median number of days until first extubation for all participants. FR were defined as those spending less than or equal to 6 days on the ventilator, whereas SR spent more than 6 days ventilated.

**Table 2:** Clinical features of participants and comparison of slow and fast resolvers

	All participants (n = 13)	Slow resolvers (n = 5)	Fast resolvers (n = 8)	*P* value
Mean age—year (range)	51.5 (29–75)	51.8 (29–67)	51.4 (33–75)	
Female sex—number (%)	7 (54%)	3 (60%)	4 (50%)	
Race—number (%)				
White	9 (69%)	4 (80%)	5 (63%)	
African American	2 (15%)	0 (0%)	2 (25%)	
Other	2 (15%)	1 (20%)	1 (13%)	
Smoking—number (%)				
Active	5 (38%)	2 (40%)	3 (38%)	
Former	4 (31%)	2 (40%)	2 (25%)	
Never	4 (31%)	1 (20%)	3 (38%)	
BMI—kg/m_2_ (range)	27.1 (15.6–38.8)	25.4 (16.9–34.6)	29.2 (15.6–38.8)	
ARDS etiology—number (%)				
Viral pneumonia	6 (46%)	3 (60%)	3 (38%)	
Bacterial pneumonia	1 (8%)	1 (20%)	0 (0%)	
Aspiration	2 (15%)	1 (20%)	1 (13%)	
Idiopathic	3 (23%)	0 (0%)	3 (38%)	
Vaping	1 (8%)	0 (0%)	1 (13%)	
PAO_2_/FiO_2_—ratio (range)	135.5 (66–250)	109.2 (67–162)	151.9 (66–250)	
Proned—number (%)	1 (8%)	1 (20%)	0 (0%)	
Paralyzed—number (%)	5 (38%)	4 (80%)	1 (13%)	
Charlson Comorbidity Index—mean (range)	3.4 (0–7)	3 (0–6)	4 (2–7)	
SOFA—mean (range)	10.2 (6–15)	10.2 (6–14)	10.1 (8–15)	
Apache II—mean (range)	21.2 (13–29)	22.6 (17–29)	20.3 (13–28)	
Days to extubation—mean (range)	10.5 (1–59)	21.4 (8–59)	3.75 (1–6)	0.0016

Age, BMI, PAO_2_/FiO_2_, Sequential Organ Failure Assessment (SOFA), Apache II, and days to extubation presented the mean value with ranges represented in parentheses. P-values compare the slow and fast resolvers using the Mann Whitney rank-sum test

### FR have a higher percentage of Tregs in their BAL than SR

BAL, tracheal aspirate, and blood samples were obtained simultaneously on day 0 and day 3, as defined in “Methods”. Tregs in the BAL, tracheal aspirate, and peripheral blood were analyzed using a panel of fluorescent antibodies and multiparametric flow cytometry, followed by standard gating to identify the percentage of Tregs (CD3^+^CD4^+^CD127^lo^CD25^+^FOXP3^+^ cells) within the CD4^+^ T cell population (Additional file 1: Figure S1, Table S1).

The data show that Tregs are present within the cell populations obtained from BAL, tracheal aspirates, and peripheral blood of patients with ARDS, as measured by the percentage of CD4^+^ lymphocytes that are FOXP3^+^ (Fig. 1). Each participant is identified by a specific symbol and further divided into slow or fast resolvers based on the median number of days to first extubation for the full cohort (Fig. 1). The percentage of CD4^+^ cells that are FOXP3^+^ averaged from the two lavages obtained at each BAL day is shown for each participant at both time points examined during their ARDS course (Fig. 1a). Importantly, the fast resolvers have a higher mean Treg value than slow resolvers (P = 0.0002). The fast and slow resolvers are significantly different regardless of day, and there is no difference between Day 0 and Day 3. The Treg percentage obtained from the individual bronchoscopy lavages (Day 0; Fig. 1b) and the mean Treg percentages for the two lavages (Day 0; Fig. 1c) are redisplayed separately to highlight differences between FR and SR. The highest Treg percentage for a participant from either of the two bronchoscopy time points also showed that FR had a statistically higher percentage compared to SR using a 2 sample t-test with a Satterthwaite correction, given unequal variances between FR and SR (Fig. 1d; p = 0.0003). In contrast, the change in mean Treg percentage from Day 0 to Day 3 (between the first and second bronchoscopy time points) showed no difference between groups, although 4 of the 5 FR for which day 3 bronchoscopies were obtained showed an increase in Tregs, whereas none of the SR increased their Tregs over this time (Fig. 1e). The same analyses of cells collected from either tracheal aspirates or peripheral blood found no difference in the percentage of CD4^+^ lymphocytes that are Tregs between FR and SR (Fig. 1f–m).

These data suggest that Tregs can be detected in the BAL compartment during ARDS and that a higher percentage of CD4^+^ cells that are FOXP3^+^ (Tregs) is associated with faster time to extubation from mechanical ventilation. This association was detected only in the BAL and not in tracheal aspirates or peripheral blood leukocytes obtained at the time of bronchoscopies.

### Two FOXP3 isoforms differ in concentration between the BAL and peripheral blood Tregs

*FOXP3* mRNA can have several splice variants in humans [22]. Two of the more common FOXP3 isoforms can vary among certain inflammatory conditions [6, 22]. To further evaluate Tregs and FOXP3 expression during ARDS, we utilized two antibody clones that distinguish two of the most common FOXP3 isoforms (Fig. 2a). Both antibodies (clones PCH101 and 150D) bind to the full-length protein, whereas clone 150D does not bind to the FOXP3 isoform encoded by the splice variant missing exon 2, part of the repressor domain of FOXP3. We found that BAL Tregs expressed more of the full-length FOXP3 isoform retaining exon 2 when compared to Tregs isolated from peripheral blood during ARDS (Fig. 2b, c). This difference was found on samples from both Day 0 and Day 3 (Fig. 2c, P = 0.0245). Cytospins of BAL or blood leukocytes were immunostained for DNA, CD4^+^, FOXP3^+^ clone 150D, or FOXP3^+^ clone PCH101. Individual FOXP3^+^ Tregs expressed both isoforms of FOXP3 (Fig. 2d, e), confirming the quantitative flow cytometric data qualitatively.

To determine if this difference in FOXP3 expression in Tregs observed in blood and lung was observed in other tissues, we searched available databases in which RNAseq was performed in simultaneously obtained blood and tissue Tregs. An analysis of RNA-seq data from Tregs isolated from normal breast tissue compared to Tregs isolated from peripheral blood was previously reported by Plitas et al. [19]. This analysis of their data showed that Tregs isolated from normal breast tissue have a greater ratio of full length *FOXP3* to a splice variant missing exon 2 compared to Tregs isolated from peripheral blood (graphed in Fig. 2f).

Alternative splicing is a common mechanism to diversify a protein’s function, and the differences in FOXP3 isoforms may suggest changes or variations in Treg functions or states in the lung during ARDS. Our data indicate that Tregs from the BAL are phenotypically different from Tregs in peripheral blood, and this mirrors changes in splicing seen in other peripheral tissue Tregs.

### Other immune cells in BAL, tracheal aspirate, and blood at Day 0 and 3 of ARDS

To characterize other immune cell population at Day 0 and 3 during ARDS in the BAL, tracheal aspirate, and peripheral blood, we employed other multiparametric flow cytometric panels and analyses (Additional file 1: Figures S2, S3, Table S1). The most numerous immune cell population identified in the BAL, tracheal aspirate, and blood was neutrophils (Fig. 3a, e, i), as defined by CD45^+^CD206^−^CD66b^+^CD24^+^ immunostaining and gating to identify select populations adapted from Tighe and colleagues [23]. In the BAL, the FR had a higher alveolar macrophage percentage, as defined by CD45^+^CD206^+^CD169^+^ expression, when compared to SR (Fig. 3b; P = 0.0477). There was no significant difference between FR and SR in the BAL at either Day 0 or Day 3 (Fig. 3c, d). There were no differences between day 0 and day 3 for any cell type in any sample type (Fig. 3a, e, i). Furthermore, we did not detect differences in any cell population between SR and FR in the tracheal aspirates or blood (Fig. 3e–l). These findings are similar to prior studies examining the kinetics of alveolar immune cells, highlighting the neutrophilic predominance in early ARDS, the worse prognosis in those patients experiencing a sustained neutrophilia in BAL fluid, and increased numbers of alveolar macrophages over time in survivors [16].

### CD4^+^ and CD8^+^ subsets in BAL and blood at Day 0 and 3 of ARDS

To understand the specific T-cell subsets during ARDS, we employed a third multiparametric flow cytometric panel and analysis (Additional file 1: Figure S4, Table S1). We evaluated the percentage of CD3^+^ cells that were either CD4^+^ or CD8^+^ in the BAL or peripheral blood at day 0 and day 3. We found that on both days 0 and 3 and in both groups (SR and FR) that the median percentage of CD8^+^ trended higher than the CD4^+^ population percentage, except for FR at Day 0 (Fig. 4a). We then determined the phenotype of each T cell population. In the BAL, the majority of CD4^+^ and CD8^+^ subsets were CD4 effector memory lymphocytes (TEM), as identified by the lack of surface expression of CD45RA^+^ and CCR7^+^ (Additional file 1: Figure S5). In the peripheral blood, the CD4^+^ TEMs were higher in percentage than the other 3 subtypes examined (Additional file 1: Figure S6), while for CD8^+^ subsets, there was a higher percentage of CD8^+^CD45RA^+^ effector memory lymphocytes (TEMRA). There was no difference between the two time points examined or between SR and FR.

### The concentration of chemokines, cytokines, and soluble mediators from BAL supernatant during ARDS identifies several differences between fast and slow resolvers of ARDS

**Fig. 5:**
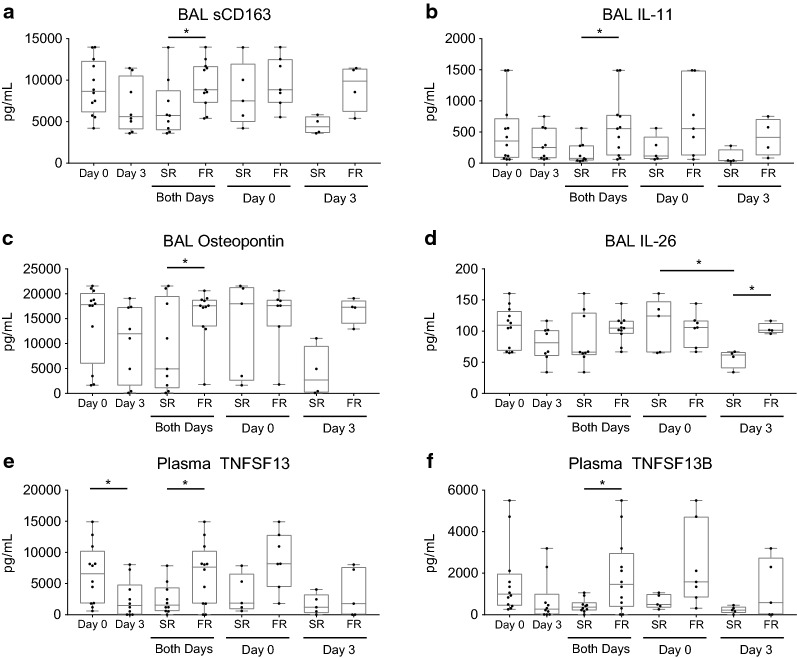
Immune mediators in bronchoalveolar lavage (BAL) or peripheral plasma from participants at either Day 0 or Day 3. Box-and-Whisker plots showing median, minimum, and maximum with overlay of individual data points (dots) of selected concentrations of mediators from Tables 3 and 4 measured in BAL or plasma at Day 0 or Day 3 after enrollment. In each figure, the data are shown for all samples, as well as when grouped as slow resolvers (SR) and fast resolvers (FR). **a**–**d** BAL concentrations of sCD163, IL-11, IL-26, and osteopontin. Day 0: n = 12, Day 3: n = 8; Combined days: SR = 9, FR = 11; Day 0: SR = 5, FR = 7; Day 3: SR = 4, FR = 5. **e**, **f** Plasma cytokine concentrations of TNFSF13 and TNFSF13B. Day 0: n = 12, Day 3 n = 10; Combined days: SR = 10, FR = 12; Day 0: SR = 5, FR = 7; Day 3: SR = 5, FR = 5. For each cell type, repeated measure ANOVA was used to compare day 0 and 3, fast and slow resolvers, and interactions. *P < 0.05

The concentrations of chemokines, cytokines, and other soluble mediators during ARDS in both the BAL supernatant and plasma from participants’ samples (12 of 13 participants) were compared at day 0 and day 3 (Tables 3 and 4; Fig. 5). In the BAL, 4 of the 37 mediators examined from all participants decreased between day 0 and day 3. Three mediators, sCD163, IL-11, and Osteopontin (OPN), were greater in concentration in FR compared to SR (Table 3; Fig. 5a–c). One mediator, IL-26, was greater in concentration in FR compared to SR at day 3, and the concentration of IL-26 decreased in SR between days 0 and day 3 (Fig. 5d).

**Table 3:** Immune mediators in BAL fluid of participants

Analyte (pg/mL)	Day 0 (n = 12)	Day 3 (n = 8)	Slow resolver Day 0 (n = 5)	Fast resolver Day 0 (n = 7)	Slow resolver Day 3 (n = 4)	Fast resolver Day 3 (n = 4)
TNFSF13	4366.9 ± 3376.7	2245.9 ± 1666.1	4188.7 ± 4860.0	4494.2 ± 2260.9	1463.44 ± 1654.4	3028.4 ± 1451.5
TNFSF13B	4556.0 ± 3347.2	1877.4 ± 1598.0	5204.0 ± 4960.8	4093.1 ± 1879.8	977.4 ± 664.0	2777.3 ± 1832.5
TNFRSF8	339.8 ± 218.5	201.5 ± 124.0	400.4 ± 324.9	296.5 ± 109.0	112.7 ± 23.6	290.3 ± 119.4
sCD163	9146.0 ± 3313.6	6855.8 ± 3156.6	8286.0 ± 3835.9	9760.4 ± 3043.7	4556.7 ± 1066.2	9154.9 ± 2831.4
Chitinase-3-like 1	14,719.3 ± 2663.3	13,691.3 ± 2672.3	15,390.7 ± 3510.7	14,239.8 ± 2035.5	13,036.8 ± 1800.7	14,345.9 ± 3503.9
gp130/IL-6RB	6604.1 ± 2809.5	4831.8 ± 2998.2	5784.4 ± 3518.3	7189.7 ± 2293.1	4154.8 ± 3480.2	5508.9 ± 2764.1
IFN-α2	*66.0* ± *23.5*	*46.6* ± *13.2*	74.1 ± 27.1	60.1 ± 20.7	40.1 ± 7.9	53.1 ± 15.2
IFN-β	126.7 ± 37.9	93.8 ± 32.1	126.7 ± 54.3	126.7 ± 26.0	77.0 ± 7.6	110.6 ± 39.9
IFN-γ	66.5 ± 28.5	50.5 ± 23.1	65.2 ± 28.7	67.5 ± 30.6	38.6 ± 11.0	62.4 ± 27.4
IL-2	95.3 ± 38.9	70.6 ± 21.7	90.3 ± 36.6	98.9 ± 42.9	58.3 ± 20.1	82.9 ± 16.9
sIL-6Rα	1495.8 ± 1426.1	872.6 ± 660.4	1998.1 ± 2196.6	1136.9 ± 389.3	526.8 ± 341.2	1218.4 ± 763.2
IL-8	7447.5 ± 5125.6	8164.9 ± 9002.5	7629.7 ± 4262.7	7317.3 ± 6000.4	13,239.5 ± 10,755.2	3090.2 ± 2181.9
IL-10	655.3 ± 1948.8	107.5 ± 72.6	1435.1 ± 3023.2	98.3 ± 28.2	81.8 ± 23.6	133.3 ± 99.9
IL-11	501.7 ± 517.4	258.1 ± 272.3	221.4 ± 211.5	701.9 ± 590.6	99.9 ± 119.9	416.3 ± 303.2
IL-12 (p40)	*104.6* ± *29.6*	*78.5* ± *27.0*	109.1 ± 37.9	101.4 ± 24.9	57.5 ± 15.1	99.4 ± 17.5
IL-12 (p70)	31.8 ± 6.4	28.9 ± 3.4	31.6 ± 7.2	31.9 ± 6.4	29.8 ± 3.6	28.1 ± 3.5
IL-19	76.2 ± 45.3	124.0 ± 189.9	92.0 ± 68.8	64.9 ± 15.6	188.6 ± 270.1	59.4 ± 6.3
IL-20	75.5 ± 26.8	53.3 ± 12.9	74.3 ± 26.3	76.4 ± 29.3	51.0 ± 17.0	55.6 ± 9.2
IL-22	78.1 ± 35.6	60.9 ± 14.4	89.6 ± 54.7	69.9 ± 12.0	64.4 ± 17.9	57.4 ± 11.4
IL-26	106.3 ± 32.6	79.9 ± 28.0	110.2 ± 42.8	103.5 ± 26.5	*56.0* ± *15.1*	*103.8* ± *8.9*
IL-27	54.3 ± 27.1	35.8 ± 7.2	54.5 ± 20.3	54.1 ± 32.8	34.4 ± 10.5	37.3 ± 1.9
IL-28A/IFN-λ2	*82.2* ± *33.8*	*49.3* ± *20.2*	82.3 ± 27.3	82.1 ± 40.0	36.8 ± 9.6	61.8 ± 21.0
IL-29/IFN-λ1	39.4 ± 13.7	30.2 ± 7.7	39.5 ± 12.8	39.4 ± 15.4	26.5 ± 4.6	33.8 ± 9.0
IL-32	111.3 ± 21.0	104.2 ± 31.2	116.5 ± 21.7	107.6 ± 21.3	114.5 ± 32.7	93.9 ± 30.3
IL-34	78.4 ± 10.9	67.0 ± 17.7	75.5 ± 7.9	80.5 ± 12.7	71.3 ± 19.6	62.8 ± 17.2
IL-35	140.8 ± 39.9	113.6 ± 20.8	139.4 ± 37.1	141.9 ± 44.8	106.5 ± 16.5	120.8 ± 24.5
LIGHT/TNFSF14	67.8 ± 12.9	66.8 ± 33.2	70.2 ± 15.1	66.0 ± 12.0	81.9 ± 42.8	51.8 ± 12.0
MMP-1	161.3 ± 103.2	101.0 ± 31.1	186.8 ± 158.8	143.1 ± 42.2	87.4 ± 33.2	114.6 ± 25.9
MMP-2	143.6 ± 76.9	125.7 ± 122.3	153.7 ± 109.2	136.3 ± 52.6	183.0 ± 158.6	68.4 ± 31.3
MMP-3	69.5 ± 33.0	52.3 ± 19.2	69.6 ± 28.5	69.5 ± 38.1	51.6 ± 24.4	52.9 ± 16.2
Osteocalcin	*108.4* ± *43.2*	*69.6* ± *16.6*	94.3 ± 21.2	118.6 ± 53.2	61.6 ± 7.2	77.7 ± 20.4
Osteopontin	14,510.9 ± 7668.3	10,381.1 ± 7674.8	13,149.5 ± 9792.5	15,483.4 ± 6420.9	4122.9 ± 5110.7	16,639.3 ± 2623.1
Pentraxin	4073.1 ± 4227.4	1865.2 ± 1638.8	3469.5 ± 2253.2	4504.3 ± 5372.0	866.1 ± 816.3	2864.3 ± 1714.2
sTNF-R1	5390.5 ± 1949.5	4198.8 ± 1573.7	5368.0 ± 2904.4	5406.6 ± 1158.9	3808.4 ± 2203.5	4589.3 ± 718.9
sTNF-R2	2085.6 ± 2343.4	934.7 ± 483.8	2687.1 ± 3666.2	1656.0 ± 768.2	775.2 ± 513.4	1094.3 ± 463.5
TSLP	261.1 ± 457.7	80.8 ± 66.7	134.0 ± 110.0	351.9 ± 594.1	39.5 ± 9.0	122.1 ± 75.8
TWEAK/TNFSF12	183.1 ± 65.1	184.2 ± 88.6	184.7 ± 62.1	182.1 ± 72.1	232.8 ± 78.6	135.6 ± 76.5

Immune mediators were measured in BAL fluid at Day 0 or 3 post-enrollment. Values are mean ± SD. The number of samples used for calculations (n) is noted in the column subheading. The immune mediators IL-11, IL-26, Osteopontin, and sCD163 demonstrated a significant difference between fast and slow resolvers (independent of day; see Fig. 5). IFN-α2, IFN-γ2, IL-12 (p40), and Osteocalcin demonstrated a significant difference between concentrations of all participants between Day 0 and Day 3 and are in italics, P < 0.05. For each mediator, repeated measure ANOVA was used to compare day 0 and 3, fast and slow resolvers, and interaction

**Table 4:** Immune mediators in plasma of participants on Day 0 and Day 3

Analyte (pg/mL)	Day 0 (n = 12)	Day 3 (n = 10)	Slow resolver Day 0 (n = 5)	Fast resolver Day 0 (n = 7)	Slow resolver Day 3 (n = 5)	Fast resolver Day 3 (n = 5)
TNFSF13	*6511.0* ± *4772*	*2539.9* ± *2986*	3366.3 ± 3113	8757.3 ± 4601	1657.4 ± 1626	3422.3 ± 3933
TNFSF13B	1630.7 ± 1725	726.4 ± 1102	631.4 ± 344	2344.4 ± 1987	233.0 ± 169	1219.8 ± 1448
TNFRSF8	889.5 ± 566	492.0 ± 465	757.5 ± 350	983.7 ± 693	497.8 ± 461	486.2 ± 523
sCD163	5729.0 ± 3578	2950.8 ± 3196	3813.6 ± 2540	7097.2 ± 3732	2189.3 ± 1468	3712.3 ± 4402
Chitinase-3-like 1	*11,140.4* ± *3988 (n* = *9)*	*5941.6* ± *5326 (n* = *7)*	9741.0 ± 6801 (n = 2)	12,301.3 ± 327 (n = 4)	2232.0 ± 3138 (n = 2)	5956.5 ± 8406.8 (n = 2)
gp130/IL-6RB	7730.6 ± 3091	4891.3 ± 4302	5633.0 ± 1964	9228.9 ± 2943	3858.6 ± 2332	5923.9 ± 5791
IFN-α2	*78.6* ± *25*	*50.8* ± *27*	74.7 ± 12	81.4 ± 32	54.8 ± 26	46.7 ± 31
IFN-β	*118.3* ± *37*	*70.7* ± *44*	106.2 ± 29	126.9 ± 42	71.5 ± 38	69.9 ± 55
IFN-γ	*105.5* ± *37*	*64.7* ± *40*	95.2 ± 18	112.9 ± 46	70.1 ± 38	59.2 ± 45
IL-2	*158.3* ± *53*	*101.1* ± *66*	154.1 ± 38	155.0 ± 62	105.6 ± 61	96.6 ± 77
sIL-6Rα	1424.5 ± 521	952.7 ± 759	1403.7 ± 796	1439.4 ± 274	874.2 ± 580	1031.1 ± 972
IL-8	*72.2* ± *23*	*44.8* ± *23*	69.1 ± 17	74.4 ± 27	48.6 ± 22	41.0 ± 26
IL-10	*105.0* ± *48*	*60.0* ± *35*	95.3 ± 23	111.9 ± 61	65.2 ± 31	54.7 ± 41
IL-11	*201.5* ± *75*	*124.2* ± *82*	193.1 ± 74	207.5 ± 82	135.0 ± 82	113.3 ± 89
IL-12 (p40)	*167.8* ± *63*	*100.4* ± *65*	152.6 ± 39	178.7 ± 77	107.9 ± 62	92.8 ± 74
IL-12 (p70)	33.9 ± 8	25.9 ± 10	35.4 ± 11	32.9 ± 7	29.4 ± 11	22.3 ± 10
IL-19	*64.9* ± *15*	*45.5* ± *21*	63.8 ± 9	65.6 ± 18	51.1 ± 20	41.8 ± 23
IL-20	*104.9* ± *37*	*66.3* ± *37*	98.9 ± 19	109.2 ± 47	71.3 ± 35	61.3 ± 42
IL-22	71.6 ± 14	51.6 ± 28	68.7 ± 9	73.6 ± 17	55.8 ± 25	47.4 ± 33
IL-26	*110.3* ± *23*	*73.5* ± *44*	104.6 ± 16	114.4 ± 27	81.0 ± 41	65.9 ± 51
IL-27	50.8 ± 14	37.5 ± 18	52.8 ± 11	49.3 ± 16	43.8 ± 19	31.2 ± 17
IL-28A/IFN-λ2	97.3 ± 37	62.6 ± 38	87.1 ± 17	104.6 ± 47	65.2 ± 34	60.0 ± 45
IL-29/IFN-λ1	51.6 ± 17	34.8 ± 19	48.2 ± 8	54.0 ± 22	39.5 ± 18	30.0 ± 20
IL-32	*113.1* ± *32*	*76.1* ± *41*	111.2 ± 25	114.5 ± 38	81.6 ± 39	70.6 ± 48
IL-34	*81.9* ± *27*	*52.7* ± *29*	80.3 ± 19	83.1 ± 33	57.8 ± 27	47.5 ± 34
IL-35	*177.3* ± *72*	*97.9* ± *65*	156.7 ± 48	192.0 ± 86	97.9 ± 56	97.9 ± 80
LIGHT/TNFSF14	*93.9* ± *45*	*47.5* ± *33*	67.4 ± 22	112.9 ± 48	47.8 ± 28	47.2 ± 42
MMP-1	*203.8* ± *71*	*119.4* ± *79*	180.9 ± 14	220.1 ± 92	128.7 ± 72	110.1 ± 93
MMP-2	1048.1 ± 1356	950.4 ± 1430	421.5 ± 200	1495.6 ± 1668	612.5 ± 791	1288.2 ± 1921
MMP-3	971.5 ± 862	715.7 ± 915	644.8 ± 441	1204.9 ± 1039	527.9 ± 539	903.4 ± 1228
Osteocalcin	339.3 ± 245	195.0 ± 209	169.1 ± 82	460.9 ± 253	162.2 ± 168	227.8 ± 261
Osteopontin	*13,693.2* ± *2460*	*8409.0* ± *6188*	12,455.5 ± 2979	14,577.3 ± 1729	9069.0 ± 5881	7748.9 ± 7106
Pentraxin	7618.4 ± 6430	2868.9 ± 2637	5515.3 ± 4217	9120.6 ± 7592	2189.9 ± 1897	3547.8 ± 3301
sTNF-R1	5070.8 ± 2934	3167.8 ± 3160	3474.5 ± 2432	6210.9 ± 2863	3450.4 ± 3366	2885.2 ± 3308
sTNF-R2	1625.5 ± 1000	934.4 ± 1062	1196.7 ± 808	1931.8 ± 1066	1046.5 ± 1128	822.3 ± 1111
TSLP	*110.5* ± *46*	*61.5* ± *39*	93.7 ± 31	122.4 ± 53	64.1 ± 35	58.8 ± 46
TWEAK/TNFSF12	149.7 ± 46	112.2 ± 76	134.5 ± 52	160.5 ± 42	109.6 ± 60	114.7 ± 98

Immune mediators were measured in plasma at Day 0 or 3 post-enrollment. Values are mean ± SD. Samples with values below the lower limit of quantitation (LLOQ) for the assay were removed from the analysis. The number of samples used for calculations (n) is noted in the column subheading, unless otherwise indicated in the individual cells when concentrations for the specific cytokine or chemokine sample fell below the LLOQ. Immune mediators in italics reached statistical significance between Day 0 and Day 3, P < 0.05. The immune mediators TNFSF13 and TNFSF13B demonstrated a significant difference between fast and slow resolvers (independent of day). TNFSF13, Chitinase-3-like 1, IFN-α2, IFN-β, IFN-γ, IL-2, IL-8, IL-10, IL-11, IL-12 (p40), IL-19, IL-20, IL-26, IL-32, IL-34, IL-35, TNFSF14, MMP-1, Osteopontin, and TLSP demonstrated a significant difference between concentrations of all participants between Day 0 and Day 3. For each mediator, repeated measure ANOVA was used to compare day 0 and 3, fast and slow resolvers, and interaction

In the plasma, 20 of the 37 mediators examined from all participants were significantly decreased in concentration on day 3 compared to day 0 (Table 4). Of these 20, two were also decreased in the BAL (IFN-α2, and IL-12 (p40)) (Tables 3 and 4). When comparing the SR and FR, only two mediators, TNFSF13 and TNFSF13B, were significantly higher in concentration in FR compared to SR (Fig. 5d, e). Figure 5 graphically illustrates the six mediators that differed between SR and FR in BAL or plasma.

## Discussion

Our study pursued the changes in immune cell populations in airways and alveoli that occur between the first 72 h of ARDS and 2–4 days later. We compared those who were extubated from the ventilator within 6 days or less with those who were ventilated for more than 6 days. We examined the changes in both myeloid and lymphoid subpopulations between two time points, as assessed by BAL performed during bronchoscopy. Our findings support the work of others, showing that Tregs are present in the alveolar space during ARDS. Moreover, the patients who spent fewer days on mechanical ventilation have a higher percentage of Tregs among their CD4^+^ T cells at both time points. These findings support the hypothesis that Tregs play a role in the resolution of ARDS, similar to what we and others have demonstrated in experimental ALI models—that Tregs play an essential role in the resolution of ALI [8, 9, 11, 24, 25].

The differentiation of fast and slow resolvers of ARDS in this study was based on the time to first extubation. This time point was chosen as it was considered to be well defined and representative of improvement in lung injury. In our institution, all patients undergo paired daily spontaneous awakening and breathing trials per protocols, and all patients in the study were extubated only after passing a spontaneous breathing trial [26]. Reasons for failure of extubation are numerous, such as persistent encephalopathy or ICU-acquired weakness. Re-intubation is frequently a consequence of these issues or other unrelated issues that can arise and be unrelated to the degree of lung injury [27]. Utilizing a standardized protocol to determine appropriateness for extubation should minimize the differences in persistent lung injury at the time of extubation. In our cohort, participant 12 was extubated 2 days after intubation following improvement in their ratio of arterial to inspired oxygen and in their work of breathing, as evidence by their rapid shallow breathing index. Unfortunately, the patient subsequently developed an aspiration pneumonitis post extubation and required re-intubation the following day. Future studies will need to consider these confounders when selecting the most appropriate study outcome metrics for resolution of lung injury.

Our data show that the Tregs within the BAL fluid are enriched for expression of the full-length isoform of FOXP3 when compared to the Tregs in the blood of the same ARDS patients. The isoforms of FOXP3 are present in humans, but mice express only the full-length FOXP3. All isoforms are functional inhibitors of CD4^+^ lymphocyte activation [28]. A recent review by Mailer discusses the alternative splicing of FOXP3 in humans in detail [22], and the mechanisms leading to alternative splice variants or their biological roles are not fully understood. One potential mechanism may be that epigenetic modifications like methylation or histone deacetylation may slow RNA polymerase II elongation, and these “closed” chromatin factors may favor full-length FOXP3 expression, while more “open” chromatin may favor exon skipping [22, 29]. In patients with coronary artery disease, increased expression of full-length FOXP3 is seen and induced by T cell receptor (TCR) stimulation [30]. Taken together, an increase in the full-length FOXP3 isoform may lead to de novo FOXP3 induction in activated CD4^+^ lymphocytes [22]. The increase in the full-length FOXP3 isoform in BAL Tregs compared to peripheral blood Tregs may suggest peripheral induction of FOXP3^+^ Treg from CD4^+^ lymphocytes during ARDS through TCR stimulation.

Of note, no serious adverse events, defined as any event resulting from a study bronchoscopy that was life-threatening, resulted in death, prolonged the hospitalization or time on mechanical ventilation, or caused persistent or significant disability or incapacity, were identified in this study. One episode of intra-procedural hypoxia occurred (defined as the SpO_2_ dropping below 90% during the procedure), necessitating early termination of the procedure, and resolved within an hour. Other bronchoscopy studies performed in patients with ARDS or other critical illness have reported similar rates of termination of the procedure due to hypoxia (range 0–2%) [31, 32]. Mild, transient hypoxia was the most frequent side effect of the procedure.

There are conflicting observations regarding the kinetics of Tregs over the course of ARDS. A study by Halter et al. examined Treg kinetics in ARDS patients at weekly intervals over three weeks and found that the bronchoalveolar Treg/CD4^+^ percentage was lower in ARDS patients than in non-ARDS patients [14]. Risso et al. showed that ARDS BAL samples obtained at an early single time point did not demonstrate differences in T cell subtypes, including Tregs, in the BAL compared to non-ARDS patients [15]. Halter et al. also reported that a higher Treg/CD4^+^ percentage in peripheral blood collected within the first week of ARDS predicted a higher likelihood of survival [14]. This study used clinical excess BAL and blood samples performed at weekly intervals as part of usual patient care. Less than half of the samples were obtained early in the course of ARDS [14]. A study by Song et al. demonstrated that Tregs as a percentage of CD4^+^ cells increase in the blood of patients with ARDS compared to healthy controls, and the patients with lung injury who survived had a greater percentage of Tregs [33]. Changes in blood Tregs may also become evident later in ARDS, and the kinetics of blood and lung Tregs are undoubtedly complex.

Importantly, analysis of Tregs within the pulmonary tissue of humans has not been performed. Evaluating Tregs in lung tissue is not possible in critically ill patients; however, Tregs within the lung tissue may be essential during the resolution of ARDS. Importantly, our work in experimental animal studies demonstrated that the vast majority of Tregs in the lung (> 99%) are not in the lavageable space and are only measured after enzymatic lung dissociation methods [8, 25]. Thus, the lavageable Tregs are likely to be the tip of the iceberg in studies of this interesting population.

Interestingly, our BAL and plasma chemokine, cytokine, and soluble mediator analysis found several mediators which distinguished between SR and FR. IL-11 concentration in the BAL was significantly greater in FR than SR (Table 3; Fig. 5b). IL-11 has been shown to play a role in platelet maturation, along with increasing the production of IL-4 and IL-5 and decreasing IL-12 production by T cells [34]. The functions of IL-11 in ARDS are unknown.

A second mediator, sCD163, was found at higher concentrations in the BAL of FR (Table 3; Fig. 5a). CD163 is a receptor found on monocytes and macrophages and functions as a scavenger receptor for hemoglobin-haptoglobin complexes [35, 36]. Interestingly, in humans but not mice, a metalloproteinase, ADAM17, can enzymatically cleave CD163 and shed soluble CD163, sCD163, which can be detected at higher concentrations in inflammatory disease processes [37]. Additionally, CD163 expression on lung macrophages may reflect either different ontogeny or different activation states [38]. In this study, sCD163 concentration was higher in FR, which contrast with other studies where sCD163 is associated with poor outcomes with patients with bacteremia [37]. The higher concentration of sCD163 in FR may reflect the greater number of alveolar macrophages that are present in FR compared to SR. It may also indicate a different regulation of shedding of sCD163 in the BAL during ARDS. For example, possible mechanisms of increased sCD163 shedding during ARDS include increased metalloproteinase activity and differences in the recruitment or activation state of monocyte/macrophage populations.

A third mediator found in higher concentration in the BAL of FR than SR was OPN (Table 3; Fig. 5c). OPN is a molecule present in the extracellular matrix of mineralized tissues but also functions as a cytokine in body fluids [39]. As a cytokine, OPN plays both pro-inflammatory roles in macrophage recruitment and early Th1 responses and anti-inflammatory roles through inhibition of iNOS and enhanced wound healing [39, 40]. In lung injury, OPN plays a role in bleomycin-induced lung fibrosis, as mice deficient in OPN develop more cystic dilated air spaces, decreased type I collagen expression, and less active TGF-β1 compared to OPN-expressing mice [41]. Alveolar macrophages highly express OPN during ARDS [42]. In African green monkeys infected with SARS-CoV-1, OPN was expressed primarily by infiltrating macrophages [43]. OPN functions to stimulate neutrophil recruitment to lungs in a murine model of transfusion-related ALI [44].

The cytokine IL-26, a member of the IL-10 cytokine family, was increased in the BAL of FR at day 3 and decreased in slow resolvers from day 0 to day 3 (Table 3; Fig. 5d). IL-26 has diverse antiviral and antimicrobial actions, and many cell types secrete IL-26 that can then signal to both epithelial and dendritic cells [45].

Only two plasma soluble mediators were found to be significantly different between SR and FR. The tumor necrosis factor ligand superfamily member 13 (TNFSF13), also known as a proliferation-inducing ligand (APRIL), and TNFSF13B, also known as B-cell activating factor (BAFF), were both expressed at higher concentrations in FR (Table 4 and Fig. 5e, f). Both molecules function in B cell development [46]. While Treg percentages were higher in the FR, B cells were similar compared to SR. In mice, Stohl and colleagues have demonstrated that BAFF concentration and B cell numbers positively correlate with the number of Foxp3^+^ Tregs in the spleen [47]. Recent data from our laboratory demonstrate that during recovery from LPS-induced ALI, Treg-depleted mice had 40% fewer B (CD19^+^) lymphocytes, again suggesting an association between Tregs and B cells [48]. The interaction between BAFF, B cells and Tregs during ARDS is not clear and will be an exciting area to pursue.

Our findings suggest that immunotherapy designed to augment Treg responses may be useful in controlling severe lung inflammation, as occurs during ARDS [49]. For instance, vasoactive intestinal peptide (VIP) has been demonstrated to generate CD4^+^CD25^+^ regulatory T cells in vivo and inhibit graft-versus-host disease in an animal model [50]. Furthermore, inhaled VIP administered in a clinical trial for sarcoidosis patients was found to significantly increase the numbers of CD4^+^CD127^−^CD25^+^ FOXP3^+^ T cells in the BAL [51]. More recently, an IL-2 mutein (a protein with an altered amino acid sequence) with reduced binding to the IL-2Rβγ receptor resulted in selective expansion and activation of Tregs [52]. Treg adoptive therapy is another potential therapeutic option, and early phase I studies have been reported in graft versus host disease [53–56]. Recently infusions of cryopreserved cord blood-derived Tregs are currently the focus of a phase 1 clinical trial for COVID-19 ARDS (ClinicalTrials.gov Identifier: NCT04468971). Selective treatment, which elicits Treg expansion or activation, or cellular immunotherapy, could be exciting possibilities for ARDS prevention or therapy in the future. While these therapies hold great potential, vigilance is needed in trials enhancing Treg numbers and function, given their potential effect on suppression of anti-tumor immunity [57, 58]. However, the short duration of exposure expected when used for treatment or prevention of ARDS, the risks would likely be low.

Our findings raise many questions regarding the role Tregs may play in ARDS. The mechanisms by which Tregs are expanded, induced, and/or recruited to the lung during ARDS are not well characterized and warrant further investigation. Indeed, if Tregs are essential for resolution of ARDS, then exploring which Treg subpopulations are essential and what are the principal mechanisms underlying Treg functions may provide insights into ARDS recovery. Leveraging Treg processes to lessen injury or accelerate recovery may be attractive opportunities for clinical studies. Understanding the successful resolution of inflammatory and immune responses is likely to have an impact on novel ways to intervene therapeutically and improve outcomes in patients with ARDS.

## Conclusions

Foxp3^+^ regulatory T cells play essential roles in immune homeostasis and repair of damaged lung tissue. Tregs are present in the bronchoalveolar compartment in humans with ARDS. Patients whose lung injury resolves quickly, as measured by time to liberation from mechanical ventilation, have a higher percentage of Tregs amongst CD4^+^ T cells in the bronchoalveolar compartment than those who resolve slowly. Tregs may contribute to the resolution of ARDS and may be a therapeutic target.

## Supplementary information

**Additional file 1.** Additional figures and tables.

## Data Availability

The datasets used and/or analysed during the current study are available from the corresponding author on reasonable request. Previously reported RNA-seq data were examined for the expression of *FOXP3* splice variants in Tregs from different sites [19]. The accession number for the published sequencing data is BioProject: PRJNA350777, and GEO: GSE89225.

## Abbreviations

APRIL: A proliferation-inducing ligand
ARDS: Acute respiratory distress syndrome
BAFF: B-cell activating factor
BAL: Bronchoalveolar lavage
FiO_2_: Fraction of inspired oxygen
FR: Fast resolvers
Foxp3: Forkhead box P3
ICU: Intensive care unit
OPN: Osteopontin
PEEP: Positive end-expiratory pressure
SpO_2_: Oxygen saturation
SR: Slow resolver
TCM: Central memory lymphocyte
TEM: Effector memory lymphocyte
TEMRA: Effector memory lymphocyte
Treg: Foxp3^+^ regulatory T cell
V_T_: Tidal volume
