# Supplementary material for: Bronchoalveolar Tregs are associated with duration of mechanical ventilation in acute respiratory distress syndrome
Source: J Transl Med. 2020 Nov 11;18:427. doi: 10.1186/s12967-020-02595-3 (PMC7656499; doi:10.1186/s12967-020-02595-3)
Supplement: Supplementary file 1 — Additional file 1. Additional figures and tables. [file 12967_2020_2595_MOESM1_ESM.docx]

**Supplemental Data**

**Supplemental Figure 1**


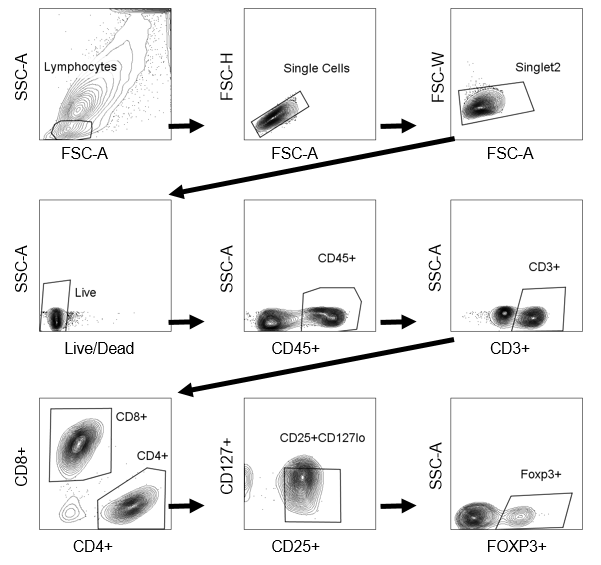


**Supplemental Figure 1. Flow cytometric gating method for identification of FOXP3^+^ regulatory T cells.** The flow cytometric plots and gating scheme used for the identification of regulatory T cells are illustrated, as described in the Methods section. Gating and dot plot results are representative of at least three independent experiments.

**Supplemental Figure 2**


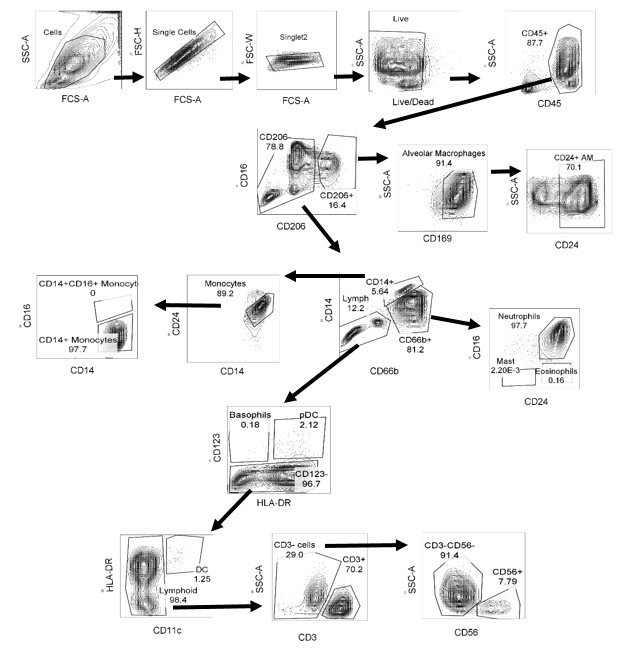


**Supplemental Figure 2. Flow cytometric gating method for identification of immune cells in the Bronchoalveolar Lavage and Tracheal Aspirate.** The flow cytometric plots and gating scheme used for the identification of immune cells are shown. Gating was adapted from (23).

**Supplemental Figure 3**


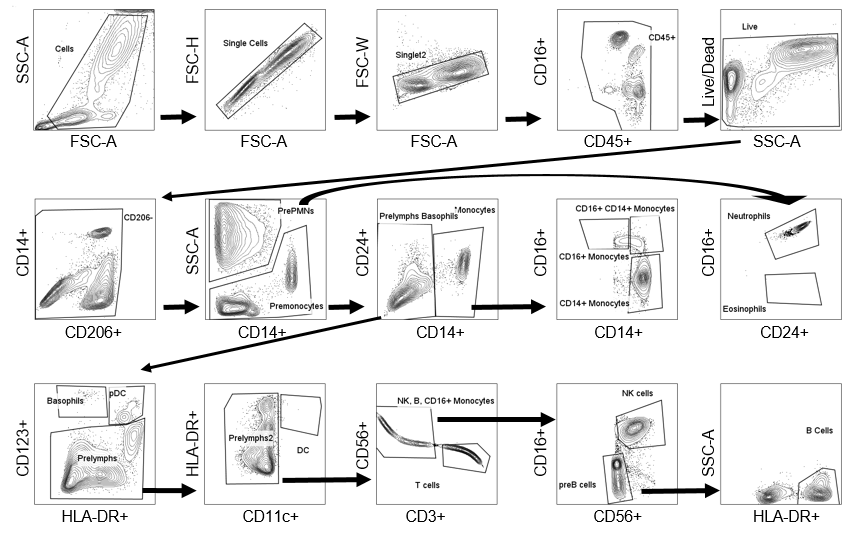


**Supplemental Figure 3. Flow cytometric gating method for the identification of immune cells in peripheral blood.** The flow cytometric plots and gating scheme used for the identification of immune cells in peripheral blood is shown. Gating was adapted from (23).

**Supplemental Figure 4**


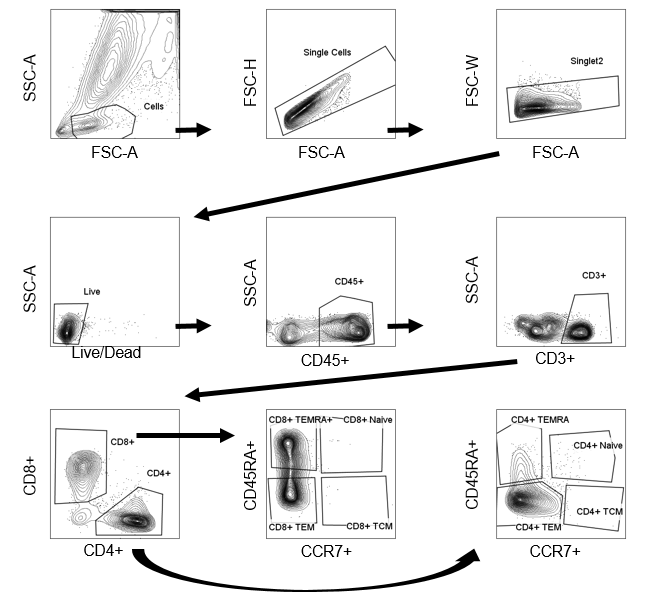


**Supplemental Figure 4. Flow cytometric gating method for identification of CD4^+^ and CD8^+^ lymphocyte subsets.** Representative flow cytometric gating of CD4^+^ and CD8^+^ cells in bronchoalveolar lavage or peripheral blood, gating for CCR7^+^ and CD45RA^+^ expression on either CD4^+^ or CD8^+^ lymphocyte to identified different CD4^+^ or CD8^+^ lymphocyte subsets. Plots are representative of at least 3 independent experiments.

**Supplemental Figure 5**

**
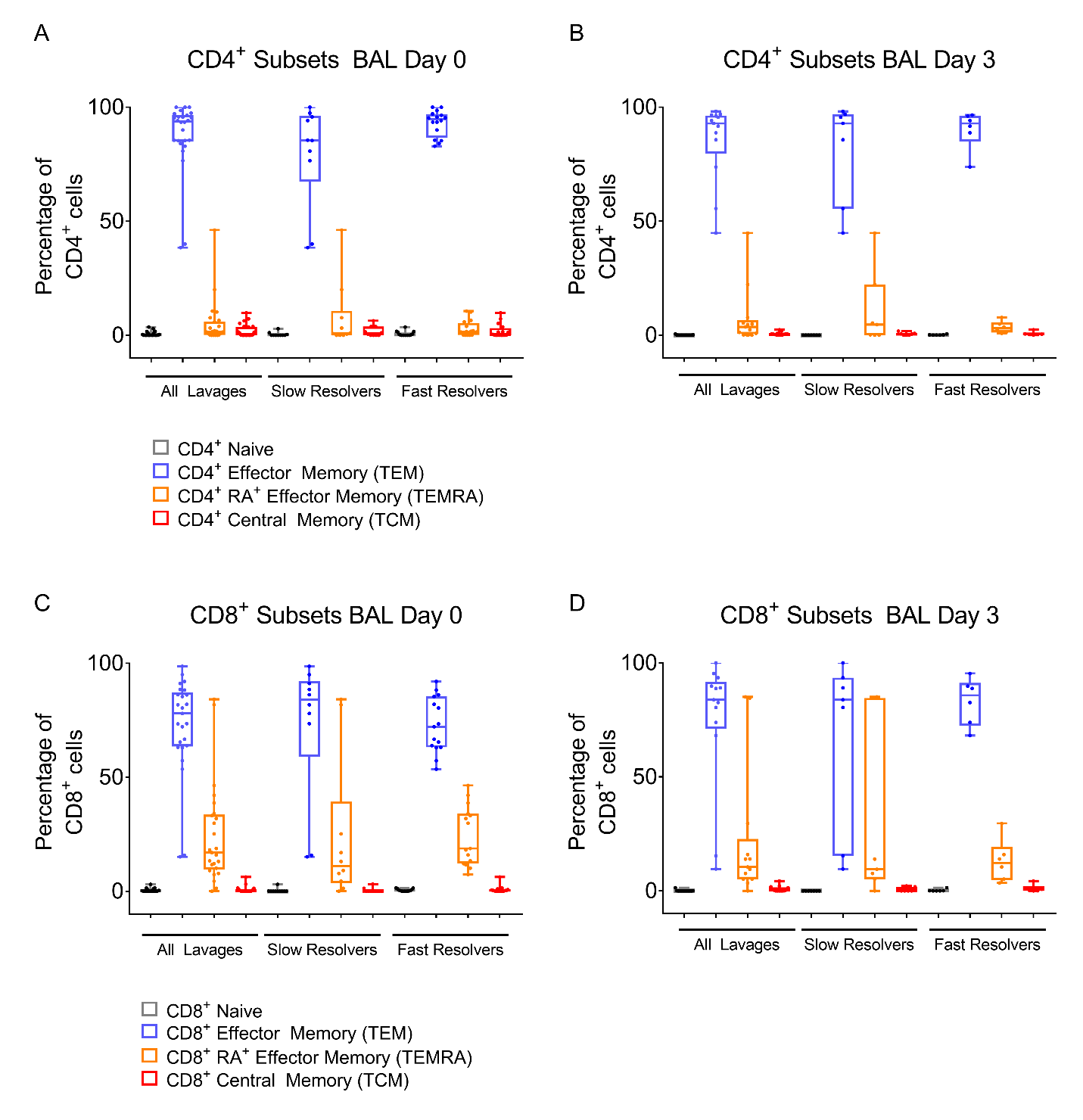
**

**Supplemental Figure 5. Percentage of naïve, effector memory, RA^+^ effector memory or central memory CD4^+^ or CD8^+^ lymphocyte subsets in BAL.** Flow cytometric gating for CCR7^+^ and CD45RA^+^ expression on either CD4^+^ or CD8^+^ lymphocytes to identify naïve, effector memory (TEM), RA^+^ effector memory (TEMRA) or central memory (TCM) CD4^+^ or CD8^+^ lymphocyte subsets. TEM: CD45RA^-^ CCR7^-^, TEMRA: CD45RA^+^ CCR7^-^, TCM: CD45RA^-^ CCR7^+^, Naïve: CD45RA^+^ CCR7^+^ The data are shown as the percentage of parent CD4^+^ or CD8^+^ population that is defined by either CCR7 or CD45RA positivity. In each graph, the percentage is shown for all lavages and after grouping as slow or fast resolvers. Box-and-whisker plots show median, minimum, and maximum with overlay of individual data points (dots). Day 0 or Day 3 samples (n = 7 – 25 lavages). (A) CD4^+^ subsets in BAL samples obtained on Day 0. (B) CD4^+^ subsets in BAL samples obtained on Day 3. (C) CD8^+^ subsets in BAL samples obtained on Day 0. (B) CD8^+^ subsets in BAL samples obtained on Day 3. No significant differences were identified. Data were analyzed for each cell type, without correction for multiple comparisons. For each cell type, repeated measure ANOVA was used to compare day 0 and 3, fast and slow resolvers, and interaction.

**Supplemental Figure 6**

**
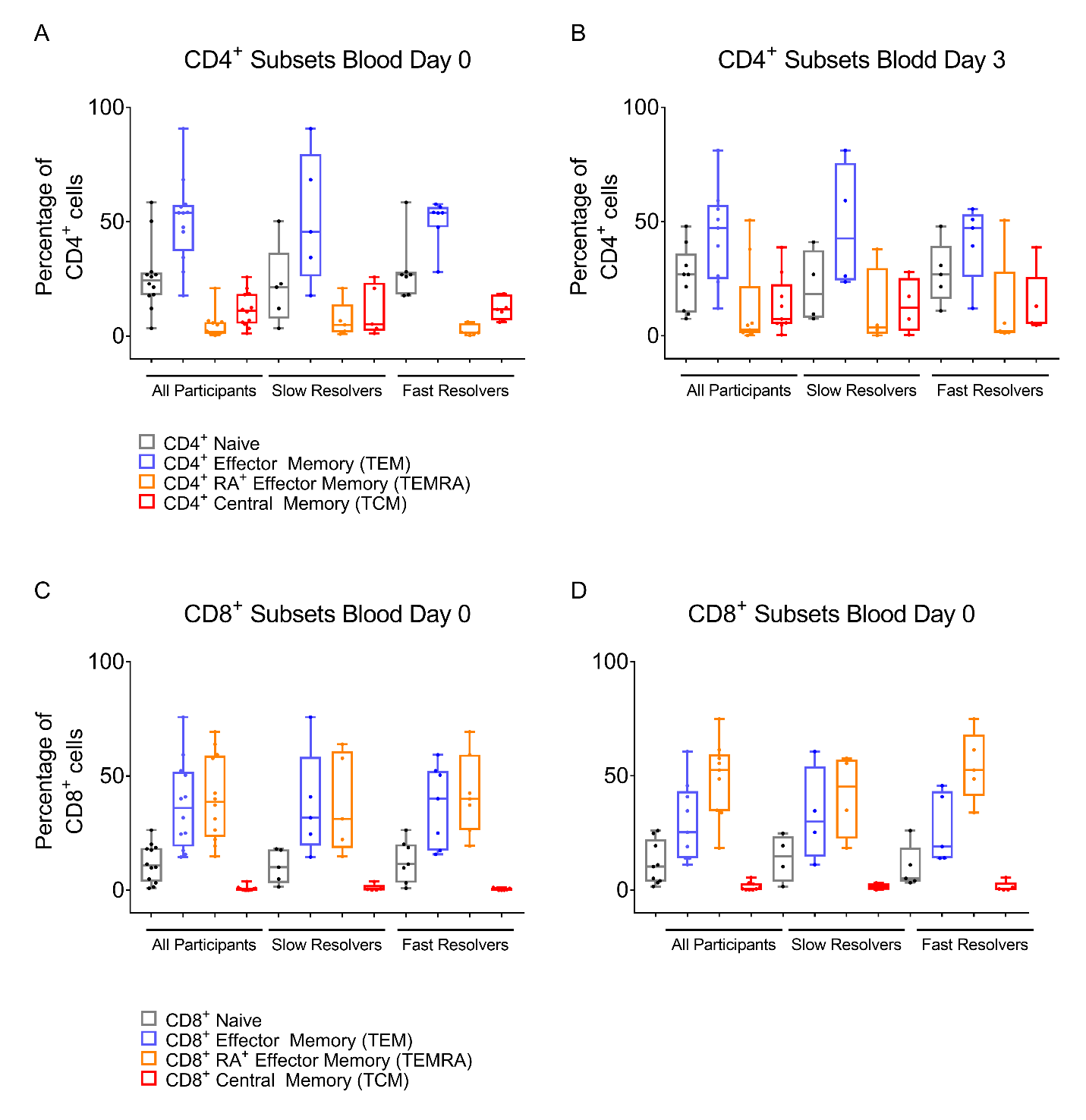
**

**Supplemental Figure 6. Percentage of Naïve, Effector Memory, RA^+^ Effector Memory or Central Memory CD4^+^ or CD8^+^ lymphocyte subsets in peripheral blood.** Flow cytometric gating for CCR7^+^ and CD45RA^+^ expression on either CD4^+^ or CD8^+^ lymphocytes to identify naïve, effector memory (TEM), RA^+^ effector memory (TEMRA) or central memory (TCM) CD4^+^ or CD8^+^ lymphocyte subsets. TEM: CD45RA^-^ CCR7^-^, TEMRA: CD45RA^+^ CCR7^-^, TCM: CD45RA^-^ CCR7^+^, Naïve: CD45RA^+^ CCR7^+^ The data are shown as the percentage of parent CD4^+^ or CD8^+^ population that is defined by either CCR7 or CD45RA positivity. In each graph, the percentage is shown for day 0, day 3 and after grouping as slow or fast resolvers. Box-and-whisker plots show median, minimum, and maximum with overlay of individual data points (dots). Day 0 or Day 3 samples (n = 4 - 12). (A) CD4^+^ subsets in blood samples obtained on Day 0. (B) CD4^+^ subsets in blood samples obtained on Day 3. (C) CD8^+^ subsets in blood samples obtained on Day 0. (B) CD8^+^ subsets in blood samples obtained on Day 3. No significant differences were identified, Data were analyzed for each cell type, without correction of multiple comparisons. For each cell type, repeated measure ANOVA was used to compare day 0 and 3, fast and slow resolvers, and interaction.

**Supplemental Table 1**

|  | **Antigen** | **Clone** | **Dilutions** | **Fluorochrome** | **Manufacturer** | **Catalog #** |
| --- | --- | --- | --- | --- | --- | --- |
| **Panel 1** |  |  |  |  |  |  |
|  | Foxp3 | PCH101 | 1:100 | APC | eBioscience | 17-4776-42 |
|  | Zombie NIR | Viability Dye | 1:50 | APC-Cy7 | Biolegend | 423106 |
|  | CD45 | HI30 | 1:50 | Alexa-700 | BD | 566041 |
|  | CD206 | 15-2 | 1:25 | FITC | Biolegend | 321104 |
|  | IFNγ | 4S.B3 | 1:50 | PE-Cy7 | BD | 557844 |
|  | CD66b | G10F5 | 1:25 | PerCP/Cy5.5 | Biolegend | 305108 |
|  | CD14 | M5E2 | 1:25 | PacBlue | BD | 552121 |
|  | CD127 | HIL-7R-MR21 | 1:25 | BV650 | BD | 563225 |
|  | CD169 | 7-239 | 1:50 | PE | BD | 565248 |
|  | CD3 | HIT3a | 1:25 | BV510 | BD | 564713 |
|  | CD4 | RPA-T4 | 1:25 | BV605 | BD | 562658 |
|  | CD8 | HIT8a | 1:25 | PE/Dazzle | Biolegend | 300930 |
|  | CD25 | M-A251 | 1:50 | BV786 | BD | 563701 |
| **Panel 2** |  |  |  |  |  |  |
|  | CD206 | 15-2 | 1:25 | FITC | Biolegend | 321104 |
|  | CD169 | 7-239 | 1:50 | PE | BD | 565248 |
|  | CD56 | HCD56 | 1:100 | PE/Dazzle | Biolegend | 318347 |
|  | CD66b | G10F5 | 1:25 | PerCP/Cy5.5 | Biolegend | 305108 |
|  | HLA-DR | LN3 | 1:100 | PE-Cy7 | eBioscience | 25-9956 |
|  | CD11c | 3.9 | 1:50 | APC | Biolegend | 301614 |
|  | CD45 | HI30 | 1:50 | Alexa-700 | BD | 566041 |
|  | Zombie NIR | Viability Dye | 1:50 | APC-Cy7 | Biolegend | 423106 |
|  | CD14 | M5E2 | 1:25 | PacBlue | BD | 552121 |
|  | CD3 | HIT3a | 1:25 | BV510 | BD | 564713 |
|  | CD24 | ML5 | 1:25 | BV605 | Biolegend | 311124 |
|  | CD123 | 6H6 | 1:25 | BV650 | Biolegend | 306020 |
|  | CD16 | 3G8 | 1:100 | BV786 | Biolegend | 302046 |
| **Panel 3** |  |  |  |  |  |  |
|  | CD69 | FN50 | 1:100 | FITC | Biolegend | 310904 |
|  | CD154 | 24-31 | 1:100 | PE | Biolegend | 310806 |
|  | CD8 | HIT8a | 1:25 | PE/Dazzle | Biolegend | 300930 |
|  | CD45RA | HI100 | 1:100 | PerCP/Cy5.5 | Biolegend | 304122 |
|  | CD62L | DREG-56 | 1:100 | PE-Cy7 | Biolegend | 304822 |
|  | CD197 | G043H7 | 1:100 | APC | Biolegend | 353214 |
|  | CD45 | HI30 | 1:50 | Alexa-700 | BD | 566041 |
|  | Zombie NIR | Viability Dye | 1:50 | APC-Cy7 | Biolegend | 423106 |
|  | CD45RO | UCHL1 | 1:50 | PacBlue | Biolegend | 304216 |
|  | CD3 | HIT3a | 1:25 | BV510 | BD | 564713 |
|  | CD4 | RPA-T4 | 1:25 | BV605 | BD | 562658 |
|  | CD127 | HIL-7R-MR21 | 1:25 | BV650 | BD | 563225 |
|  | CD25 | M-A251 | 1:50 | BV786 | BD | 563701 |
| **Panel 4** |  |  |  |  |  |  |
|  | Foxp3 | 150D | 1:50 | PE | Biolegend | 320008 |
|  | CD8 | HIT8a | 1:100 | PE/Dazzle | Biolegend | 300930 |
|  | CD45RA | HI100 | 1:100 | PerCP/Cy5.5 | Biolegend | 304122 |
|  | Foxp3 | PCH101 | 1:100 | APC | eBioscience | 17-4776-42 |
|  | CD45 | HI30 | 1:50 | Alexa-700 | BD | 566041 |
|  | Zombie NIR | Viability Dye | 1:50 | APC-Cy7 | Biolegend | 423106 |
|  | IL-10 |  |  |  |  |  |
|  | CD3 | HIT3a | 1:25 | BV510 | BD | 564713 |
|  | CD4 | RPA-T4 | 1:25 | BV605 | BD | 562658 |
| **IF** |  |  |  |  |  |  |
|  | CD4 | RPA-T4 | 1:100 | Alexa Fluor 488 | Biolegend | 300519 |
|  | Hoechst 33342 |  | 1:100 |  | Fischer | H3570 |
|  | Foxp3 | 150D | 1:50 | PE | Biolegend | 320008 |
|  | Foxp3 | PCH101 | 1:100 | APC | eBioscience | 17-4776-42 |
| **Fc Block** | Fc Block | TruStain FcX | 1:50 | - | Biolegend | 422301 |

**Supplemental Table 1.** Fluorescent-conjugated antibodies or stains used in this study. IF = Immunofluorescence.

**Supplemental Table 2**

| **Excitation** | **Fluorescence Channels** | **Fluorochromes** |
| --- | --- | --- |
| 488 nm | 525/40 BP | FITC |
|  | 585/42 BP | PE |
|  | 610/20 BP | PE/Dazzle |
|  | 690/50 BP | PerCP-Cy5.5 |
|  | 780/60 BP | PE-Cy7 |
| 637 nm | 660/20 BP | APC |
|  | 712/25 BP | Alexa 700 |
|  | 780/60 BP | APC-Cy7 |
| 405 nm | 450/45 BP | PacBlue or BV421 |
|  | 525/40 BP | BV510 |
|  | 610/20 BP | BV605 |
|  | 660/20 BP | BV650 |
|  | 780/60 BP | BV786 |

**Supplemental Table 2.** Cytometer laser profile, bandpass filters, and respective fluorochromes used for each channel.
